# Planning for the Discharge, not for Patient Self-Management at Home – An Observational and Interview Study of Hospital Discharge

**DOI:** 10.5334/ijic.3003

**Published:** 2017-11-13

**Authors:** Maria Flink, Mirjam Ekstedt

**Affiliations:** 1 Department of Learning, Informatics, Management, and Ethics, Karolinska Institutet, Stockholm, SE; 2 Functional Area of Social Work, Karolinska University Hospital, Stockholm, SE; 3 School of Health and Caring Sciences, Faculty of Health and Life Sciences, Linnaeus University, Kalmar, SE

**Keywords:** care transition, discharge encounter, discharge letter, self-management

## Abstract

**Introduction and objective:**

Despite recent interest in care transitions, little is known about how patients are prepared for the self-management tasks following the hospitalization. The objective of the study was to explore how discharge information is prepared and provided to patients in the transition from hospital to home.

**Method:**

The discharge process at three hospitals in Sweden was observed over 12 days spread over ten weeks. In total, 30 discharge encounters were observed followed by interviews with patients and professionals. Data were analysed using qualitative content analysis.

**Results:**

Much time, effort and resources were used to prepare the discharge; home-going teams and registered nurses planned the practical and social aspects of the discharge and the physicians compiled a plain-language discharge letter. Less focus was given on the actual discharge information to the patients. The discharge encounters lasted for a median of 4:46 minutes and the information had a retrospective focus with information on the hospitalization period, though omitting self-management tasks and life-style advice.

**Conclusion:**

The discharge letter constitutes the basis for all patient information at discharge. The focus of the discharge encounter needs to be extended beyond mere information to include patient understanding, motivation and skills for self-management at home.

## Introduction

In recent years, much attention has been given to care transitions between healthcare settings and to re-hospitalizations [1,2,3]. This is not without reason, as errors in medication, therapy, and follow-up of tests following hospitalization are common [4,5,6]. For most patients, and especially patients living with chronic conditions, the care transition from hospital to home marks the beginning of a new round of self-management activities. Self-management includes a patient’s abilities ‘to manage symptoms, treatment, physical and psychosocial consequences and life-style changes’ [7] that follow the illness. Healthcare systems have much to gain from facilitating patient learning about self-management and engaging patients to become active partners in care [8], as patients with high levels of activation have the most effective self-management skills [9,10,11]. Additionally, those with high knowledge of both their disease and its management have shown lower re-hospitalization rates [12]. As the risk for re-hospitalization is highest during the first weeks at home [13], the discharge encounter is a crucial occasion to collaborate with patients on their needs for effective self-management at home.

Despite years of working to involve patients in management of their own care, either through legislation and targeted healthcare reforms [14,15], or through the multiple programs focusing on improving care transitions and discharge planning [3,16,17], such efforts are still far from integrated into daily care. Commonly, patients do not seem to be prepared for this essential part of disease management upon hospital discharge [18,19]. Accordingly, patient understanding of medications, diagnosis and follow-up appointments post-discharge is deficient [20,21,22]. More than half of discharged patients could not recall the follow-up appointments that were planned post-discharge [20]. Interview comparisons of patient medication lists at discharge and patient perceptions of their medication have shown that patients did not know the names of 81.6% of their stopped medications, could not name medications and doses for 64% of their new medications, and were unable to name the medication and changed frequency in 69.3% of their re-dosed medications, even though they were allowed to check their discharge instructions during interviews [22]. During the hospital discharge process, patients are supposed to be provided with the information needed to understand both what has happened during their hospitalization and what will happen post-discharge [23]. However, it has been reported that patients are unsure of whether they actually even had a discharge encounter [24,25].

This raises questions over the weak links in the work processes that concern transition from hospital to home; how multi-professional teams prepare and execute patient discharge and how the discharge information targets patient self-management at home. Hence, the objective of the study was to explore how the discharge information is prepared and provided to patients in the transition from hospital to home.

## Material and methods

An exploratory, qualitatively driven study using observation methods and interviews was conducted. Observational techniques were used to record events and communications as they occurred and supplementary interviews were used to elicit descriptions and clarifications of actions embedded in the observed situations that could not easily be understood (such as motivations and thoughts).

Permission was obtained to observe the procedures of discharge at three different locations: the medical wards of one regional, one general, and one university hospital, all publically financed and located in the county of Stockholm, Sweden. These locations were selected to get a range of work organizations, discharge routines, staffing and patients with different sociodemographic backgrounds. All wards contained single rooms, double rooms and four-bed rooms and were staffed with multi-professional teams.

Multiple data collection sources were applied: *observations of encounters* in the discharge process (i.e., pre-round meetings, bedside rounds, and discharge encounters) and *shadowing of professionals’ work*, including informal conversations with patients, registered nurses (RN), aid nurses and physicians between encounters to clarify the observed discharge processes, and semi-structured *interviews* with patients, RNs, aid nurses and physicians involved in the discharge process. The focus of data observation was tasks and places where the patients’ discharge was prepared or executed, including meetings, interactions and actions during the day of discharge. Next-of-kin who were present at the discharge encounter participated in the interviews, but were not specifically targeted with questions. Semi-structured interview guides were used to get patients’ and professionals’ experiences of the discharge encounters and the discharge information. The professionals were also asked how they perceived patient understanding, and what information that is most crucial to give patients. The patients were asked how they perceived the information that the professionals gave, how sufficient the information was and what was needed for them to effectively self-manage post-discharge. Observations and interviews were tape-recorded and transcribed verbatim. Field notes were taken at formal and informal meetings and events not tape-recorded due to patient or healthcare professional non-consent. In total, 31 patients were either interviewed, observed or both. Ten of these patients had pulmonary diseases, 12 had cardiovascular diseases, and nine had various conditions. The patients were aged from 18 years to 90 years, with nine patients younger than 65 years old. An overview of the data collection is presented in Table 1.

**Table 1:** Overview patient data collection.

	Patient interview	Observation physician discharge	Physician interview	Observation RN discharge	RN interview	Coordinator interview	Aid nurse interview

University hospital	7	10	6	1	2	1	0
Regional hospital	7	14	6	2	1	0	1
General hospital	3	3	2	0	1	1	0
In total	17	27	14	3	4	2	1

RN = Registered Nurse.

Data were collected over a ten-week period, during 12 daytime shifts, generally between 8.30 AM and 3.30 PM. These hours were chosen as most patients are discharged in the daytime at the selected wards. Data were collected by three researchers – one social worker and two RNs – of which two were the authors of this article. All were experienced with the methods and have rich experience with inpatient care, though none had worked at the wards studied. During one day, a care coordinator who was part of a home-going team was shadowed and her routines observed. During the remaining 11 days, the researcher started the day by shadowing an RN or an aid nurse in their morning routines until the first pre-round meeting. The researcher observed the pre-round meeting and the rounds, including the informal communication in the waiting for all professionals to arrive. The researcher thereafter either observed the physician or the RN preparations before the discharge and the discharge encounters, as well as made the follow-up interviews. Three to four discharge encounters were observed each day. The researcher joined the professionals for lunch and coffee breaks. Informal conversations with professionals, patients and next-of-kin were conducted in the time between the regular events of the day. Data were collected until sufficient information power [26] of the studied phenomenon was considered to be achieved. This concept provides a framework to inform the decision of ending data collection. Our decision was based on the narrow study aim and high sample specificity, i.e., that all observations and interviews targeted a known, clearly defined phenomenon in which all informants had deep and broad experience.

All data, i.e., observations and semi-structured interviews, were analysed by the two authors using qualitative content analysis inspired by Graneheim and Lundman [27] to structure the coding. In this, all data (field notes, transcripts of observations and interviews) from each hospital were analysed separately and then compared. First, data from the university hospital was divided into meaning units which were condensed, and thereafter coded by the two authors. The coding was discussed until consensus was achieved. During coding, data (interviews from different stakeholders, observations, field notes) were given coloured marks to ensure that their origins were not lost. The codes were sorted into categories. Second, data from the regional hospital were analysed the same way, and then data of the general hospital were treated similarly. The categories were thereafter compared, searching for differences and similarities across categories in the different hospitals and data origins, and merged to themes (Table 2). Third, patient cases that illustrated the themes were selected.

**Table 2:** The data analysis process.

Categories	Sub-themes	Themes

The role of the emergent healthcare setting to facilitate patient self-management	Discharge letters in the emergent healthcare setting	Preparations before discharge to join pieces of information
Organizational aspects affect the professionals’ discharges		
The need for informal conversations when formal meetings are lacking	Joining information in formal and informal meetings	
Informational collaboration of the team		
The prerequisites of the individual patient to comprehend information of self-management	Patients’ own prerequisites for self-management understanding	Structured information, struggle with understanding
Understanding information requires engagement		
Patient discharge without ensuring understanding of self-management	Healthcare professionals’ efforts to ensure patient self-management understanding	
Understanding not targeted during hospitalization, despite opportunities		
Professionals make sure patients understand information		
Clarity and pedagogical tools		

The study was approved by the Regional Ethics Board in Stockholm, Sweden: no. 2014/1498-31/2.

## Results

The following results cover two themes: ‘Preparations before discharge to join pieces of information’ and ‘Structured information, struggle with understanding’.

### Preparations before discharge to join pieces of information

The decision to discharge a patient was made through a two-step meeting procedure: a pre-round meeting and a bedside round. Each pre-round meeting included senior and junior physicians, RNs, aid nurses and in some cases occupational therapists or physiotherapists. In such meetings, at which patients did not attend, the professionals’ different perspectives were discussed to get a joint perspective on the patient’s status and situation as a basis for decisions on a future care plan and discharge. The physicians, RNs and aid nurses had rotating schedules, which meant that patients met new physicians and RNs every, or every second day. During the bedside round, the patient was informed about this joint perspective, and the decision to discharge.

The wards differed partly in their preparation procedures. In one of the wards, the pre-round meetings were held in the hall outside the patients’ rooms due to a lack of an administrative room that could fit all involved persons. In this ward, the meetings were short and centred around the physicians’ questions to the RNs about patient status. The RNs described in interviews that they limited information to the physicians on patients’ personal issues, since other patients could overhear the pre-round meetings. The other wards had shared administrative rooms for physicians, RNs and aid nurses. Their meetings, which generally took more time, had a more informal character. In addition to such meetings, one ward also had a weekly meeting regarding patients who had been hospitalized for five days or more. In these meetings, all involved healthcare professionals had to give their perspective on what could be done for the patient to ensure prompt discharge. In no cases were patients involved in the pre-round or weekly meetings.

The decision to discharge was in all observations made on the same day as the actual discharge took place, giving both patients and professionals only a short time for preparation. The professionals were under pressure to complete discharge before lunch or early afternoon to make room for patients waiting in the emergency room. RNs described that they tried to start preparing for the discharge as soon as patients were admitted to the ward, for example by asking about the home situation to identify need of home-help service and taking the necessary actions in time. Patients, on the other hand, had limited time to mentally prepare for the discharge as the decision was given during rounds, i.e., generally 1–3 hours before the actual discharge. None of the observed or interviewed patients had prepared questions for the discharge encounter. An information brochure on discharge was provided in one of the wards, but no patient used it or referred to it in the discharge encounters: “I have looked at it, but I have not done anything about it.” [interview, woman 75 years, atrial fibrillation]. The short time between decisions and discharge encounters also limited the next-of-kin’s possibilities to be present and prepared for the discharge encounter. In the case of Anna (see **Box 1**) the presence of the next-of-kin was an important part of the successful discharge experience. In two of the cases where next-of-kin were present, they functioned as interpreters, which limited their ability to be active participants in the communication.

**Box 1: Patient case Anna.**

Anna, a woman aged 66 years, recently diagnosed with chronic obstructive pulmonary disease, hospitalized overnight, is very worried about her diagnosis and how to manage at home. Anna has asked her son, working full-time, to come to the hospital for the discharge encounter. The son describes that he is stunned that so many physicians treat his mother as if she is mentally incapable and do not understand that she is simply worried about how she will manage her illness at home. The resident physician describes that for patients with this kind of anxiety problem it is crucial to be well-prepared, to give thorough information and to bridge the contact to the next healthcare provider. In addition to the usual written information in the form of a discharge letter and medication list, the physician has also prepared by bringing copies of the patient’s lung x-ray and lab results. When the physician enters the patient’s room for the discharge, the son has not yet arrived. The physician decides to wait for the son. The physician walks the patient and the son through the written information carefully, step-by-step. The patient will be followed-up by a general practitioner. The patient has had bad experiences of the primary healthcare centre and asks for information to bring to the general practitioner. The physician promises to check if the patient’s primary healthcare centre can access the hospital’s electronic records and if not, to bring all necessary copies to the patient. The practical issues (need of walking aid, arranging transportation home) as well as a re-check on patient understanding of medication list are managed by the registered nurse and the physiotherapist at the ward. Both the patient and the son are very pleased with the discharge.

The RNs used a special note called “discharge planning” in the electronic medical record to facilitate the rotating RNs’ overview of discharge plans. All wards had coordinators (RNs or aid nurses) who were responsible for preparing social and practical issues related to discharge and admission, for example arranging home-help services. One of these wards also had a “home-going team” with RNs, occupational therapists and physiotherapists. The RNs described that the coordinators saved time for them in their daily work. Disadvantages with having a coordinator were also described; as the patient-responsible RN did not have an overview of the patient’s social and practical needs. One RN with more than ten years’ experience expressed concerns that the more recently graduated RNs would miss out on learning to take the patient’s social perspective into account.

When all bedside rounds were completed, right before the discharge encounter, the junior physician (or the senior physician, when no junior physician was on duty) wrote the discharge summary for the patient record. The physician also wrote a plain-language discharge letter that was given to the patient at the discharge encounter. The discharge letter followed a template which included headings on medical history, tests and treatments during the hospitalization period, medication changes and information on follow-up post-discharge. It was described as a difficult and time-consuming process to write this letter in an understandable yet correct way. One junior physician was observed struggling to find the plain-language word for ‘ischemia’, but left out the information when he failed to do so. The RNs wrote a separate discharge summary of nursing activities, but this was not given to the patients. The RNs transferred this information to RNs at nursing facilities but not to RNs in primary healthcare. The communication with RNs in primary healthcare was mandated through a web-based communication tool not compatible with the electronic medical record.

### Structured information, struggle with understanding

The physician discharge encounters ranged in time from 1:13 to 28:03 minutes, with 4:46 minutes as the median. Five discharge encounters were shorter than 3 minutes. In these cases, two patients had had informative bedside rounds just before the discharge encounter and one patient with cancer was going to meet her oncologist for an extra meeting right after the discharge encounter. The other two patients had severe problems understanding the information, due to hearing and cognitive impairment. In these cases the physician briefly described the content of the discharge letter and then directed the patients to the discharge letter if they or their non-present next-of-kin had any questions. In the longest encounter (28:03 minutes), the junior physician let the very talkative patient relate her personal situation and history. The second longest discharge encounters (17:53 minutes) included interpretation through a telephone service, which prolonged the encounter. The content of physicians’ information in these discharge encounters did not differ from that in the shorter encounters with other patients.

In all three wards, the RNs and the physicians had separate discharge encounters, although the RNs occasionally also joined the physicians in their discharge encounters. No differences on how information was given were found between the wards. Differences were found between individual professionals. The RNs had more informal talks, usually standing up by the bed-side. They focused their information on the practical details related to the discharge, e.g., information on home-help services; transportation to home and making sure family members and home-help services had received information about the discharge. The physicians often sat down by the bedside for the discharge encounters. The patients got oral information and the plain-language written discharge letter together with a medication list. The discharge information generally followed the order of the discharge letter template. The information was concluded with self-management activities in the form of medication and follow-up appointments. The template of the discharge letter did not include headings on symptom control and lifestyle advice, which were therefore commonly omitted. Oral information on symptom control was general and sparse: “if you get worse you should come back (to the hospital)” [physician discharge of man, 64 years, chronic obstructive pulmonary disease], with no clarifications on what ‘get worse’ meant in terms of severity or frequency of symptoms. Examples of specific instructions were also sparsely observed, such as “if you are really ill, if breathing gets very difficult” [physician discharge of woman, 72 years, heart failure]. Communication about lifestyle behaviour found in six discharge encounters was restricted to one-way communication that did not invite patients to participate: two patients with myocardial infarction were advised to go for walks, with one patient also told that: “you ought to lose weight” [physician discharge of man, 43 years, chest pain]. One patient with chronic obstructive pulmonary disease was asked if he smoked, but denied doing so. Three patients (pulmonary diseases and myocardial infarction) took the initiative to ask if they were allowed to exercise. No patients received referrals, tools or advice on how to manage lifestyle behaviour or changes.

The healthcare professionals were well aware that the discharge encounter was not an ideal situation to facilitate patient understanding of healthcare information. The professionals described that the patients were too stressed or too focused on going home to be able to grasp all the information. It was considered especially difficult to ensure understanding for older persons who were too polite to ask questions or for persons with multiple, complex conditions, where the information was more complex. Both RNs and physicians described in the interviews that changing health-related behaviours and giving self-care information was important, but that it was not a task that could be managed entirely by them in an emergent hospital setting.

“I really try to take responsibility to make the patients understand (their medications). Information is important, but reminders are necessary, and they are not possible to give at a short-term ward” [physician interview].

Several examples of initiatives to help patients understand were found: the professionals walked the patient through the medication list step-by-step, holding the list so that the patient could visually follow the steps; handwritten notes or markers on medication lists were used to highlight important information; the use of ‘teach-back techniques’, i.e., the patient was asked to repeat the given information; short pauses to allow the patient to read through the written material; and skills training in medication administration, as in the patient case Bertil (see **Box 2**). However, examples were also found of the professionals realizing that the patients did not understand the information, but no efforts were made to send referrals to primary healthcare or to interfere beyond giving information, as in the patient case Carl (see **Box 3**).

**Box 2: Patient case Bertil.**

Bertil, a man aged 73 years who only understands/speaks a few words in Swedish, has been hospitalized for almost a week after a myocardial infarction. The discharge is conducted using an interpreter via speakerphone. The resident physician informs the patient about his diagnosis, treatments conducted and the post-discharge medications. During the discharge encounter, the physician asks five times in various phrasings if Bertil understands the information; he confirms that he does. The registered nurse and the aid nurse wait outside the discharge room, concerned about how the patient will manage at home. The physician describes her worries. The registered nurse suggests that the physician should write a referral to the primary healthcare centre to help the patient with the medications. The aid nurse suggests that the volunteer Red Cross personnel (available during daytime to assist patients at the hospital) can help collect Bertil’s medications at the hospital pharmacy and buy a medicine container, to make sure he gets his medications before he is summoned to the primary healthcare centre. The physician agrees, and asks the registered nurse to help the patient place his medications in the container before discharge. When Bertil is back with the container and the medications the registered nurse shows him step-by-step how to read the medication list and put the right medication in the right box. The registered nurse first shows the procedure and then lets him try a few times under her supervision. She then leaves him for a few moments to get a missing medication from the storage at the ward. When she gets back the patient is sitting in bed, not knowing how to proceed. The registered nurse helps the patient fill the rest of the container. The physician and aid nurse wait in the hall outside to get the registered nurse’s view on patient understanding. They all agree that a referral must be sent to the primary healthcare centre.

**Box 3: Patient case Carl.**

Carl, a man aged 82 years, diagnosed with heart failure, was hospitalized two days due to fainting at home. The medication list noted at admission does not correspond to the medication list described by the patient at the discharge encounter. The physician has removed one medication that was thought to contribute to the patient’s fainting spell. However, Carl says at discharge that he has never taken this medication. Carl is convinced that he has too many medications overall and that this is one of the reasons why he is ill. He only takes the medications that he considers to be important and only when he feels ill. The physician goes through the medication list, to sort out which medications the patient is actually taking. The physician tries to convince the patient to take important medications, for example Trombyl, by saying that it is needed for his heart problems. Carl on his hand argues that “It is hopeless, taking 6 to 7 medications. I get all tired out. I take what is important. I think it is enough. I feel okay.” After a while the physician gives up and ends the encounter by stating that the patient can discuss his medications with his heart specialist. Afterwards, the physician describes that the hospitalization was more or less in vain, as the admitting physician had not checked the admission medication list with the patient’s actual usage. The patient on the other hand feels content with the discharge encounter. “I stood my ground, because I eat too many medications.”

The use of both oral information and written information (the discharge letter and medication list), was described as facilitating understanding, as the patients received the same information twice, and could reread and share the information at home. According to RNs and aid nurses, patients commonly phoned or showed up at the ward after discharge, to ask questions related to the hospitalization, despite having received information. This was considered complicated, since there was little time to deal with those patients, and the responsible physician was often not available. The professionals expressed in several cases an uncertainty regarding if the information was understood. “I give the information to the patients at discharge. And then I hope that they’ll read through it and think it over once they’re at home. I hope that the message is clear enough that they can understand it later.” [physician interview].

All interviewed patients except one described that they were content with the information they had been given at the discharge encounter. However, several patients added that it was difficult to think of questions during the encounters, and that the questions often arose after the discharge, when they got home. Patients also stated that thanks to their pre-understanding, they could follow the information given at discharge: “Interviewer: Was the information you got sufficient? Patient: Yes, since I knew about the medications from before”. [man with chest pain, 43 years].

## Discussion

This study addresses how discharge information is prepared and provided to patients, and how healthcare professionals provide information to patients. The discharge process was an ongoing process in several rounds including a pre-round meeting, a bedside round, writing of information and an actual discharge encounter. The preparation phase of the discharge information differed between settings, with professionals at two wards having more informal discussions before patient discharge. However, the difference in the delivery of information was more obvious between different professional groups (nurses and physicians), and in individual communication styles, than between the wards.

The discharge letter constituted the basis of the physicians’ discharge information, as both the oral and written information followed the template of this letter. The importance of writing understandable discharge letters is well-known [28]. There is evidence that information alone does not promote patient adherence to medication prescriptions [29] and that self-management interventions targeting a combination of educational, behavioural and affective components provide the most beneficial support for patient adherence [30]. This is also supported by for example the Information–Motivation–Behavioural Skills Model [31] which acknowledges that patients will be likely to initiate and maintain health-promoting behaviours if they have received the required information and are also motivated and have the essential skills for active self-management of their own care. However, our findings indicate that the hospital discharge process focused more on providing information and rarely on ensuring patient motivation or skills, which is also confirmed in a recent multi-site study [32]. Especially patients with low levels of activation [9] could be helped to more effective self-management if the goal of the discharge process could be shifted from merely provide information to ensure patient understanding, motivation and skills. Rather, healthcare professionals are challenged to reach a common ground of understanding [33] with the patient, as a basis for patient activation and engagement in making daily decisions that affect their health. Still, there seems to be a lack of use of evidence-based tools and methods, similar to what has been developed for facilitating professionals’ shared understanding in handover situations (e.g., SBAR [34] or the like). The initiatives to facilitate patient understanding (e.g., use of teach-back or pausing to allow for patients to read information) seemed more related to individual professionals’ styles than to an explicit strategy to promote patient understanding. For example, the use of teach-back is a well-known effective method to promote patient understanding [35]. Giving patients the time to reflect, the principle of ‘wait time’ or ‘three-second pause’, is another method used in pedagogical teaching [36]. Awareness of the potential of such simple tools, and conscious strategies of how to use them could potentially increase patient engagement in encounters and enhance understanding.

Much time, resources and efforts were put into planning the discharge, including practical details, as well as preparing the plain-language discharge letter, which is time well-invested; since this has been shown to decrease both readmission and length of stay for patients with medical diagnoses [17]. The plain-language discharge letter could, despite the effort associated with it, be an important way to raise patient understanding of information [37]. However, as the discharge letter had a retrospective focus overall, leaving out information on self-management activities going forward post-discharge, the opportunity to increase patient understanding, motivation and skills is limited. A recent large interview study of readmitted patients confirms in its conclusions that more clarity in discharge instructions is needed [25]. The findings indicate that the hospitalization was not part of an integrated care context. The plain language discharge letter did not draw upon patients’ care history or integrate any prospective care plans or care contacts that bridged the patient’s care episodes.

In contrast to the time spent on planning the discharge, the execution of the actual discharge encounter was not given much time (median 4:46 minutes). Ensuring that the patients actually do understand the discharge information and the self-management activities needed is difficult and time-consuming. The responsibility between primary and secondary care in performing this task is unclear, even though it is the responsibility of the discharging physician to assess the patient’s ability to perform self-management post-discharge according to Swedish legislation. However, hospital units are rarely evaluated or reimbursed for proactive initiatives that pay back in a distant future, meaning that making sure that the patient actually understands the information on self-management tend to be forwarded to someone else. Bridging activities such as post-discharge telephone calls could potentially be effective; if not to decrease readmission [38,39], at least to increase patient engagement and satisfaction [38,40]. However, several effective care transition and discharge planning interventions in recent years [3,16,17] include extra persons (e.g., transition coaches, nurse case managers, pharmacists), which reveals the difficulties embedded in these tasks. To really achieve change and meet the challenge that patients increasingly are expected to manage their self-care at home, integrated support efforts are needed, ranging across inpatient care, home healthcare and primary care. The development of care plans spanning across the care continuum could clarify the role of the stakeholders involved in patient care. Sweden has a long tradition of electronic healthcare records that are shared between primary and secondary care, providing a functional integration of services that could facilitate the development of care plans. Despite the efforts associated with developing care plans, this could potentially both ease the burden of the writing of plain language discharge letters and, more importantly, improve the transition between care settings.

## Methodological considerations

This study has several strengths and limitations. We employed multiple methods to verify the trustworthiness and enhance the quality of the findings. We triangulated the patient discharge process from the points of view of the different healthcare professionals involved as well as of patients, in three different settings. However, we did not observe the entire hospitalization period for the patients included. This means that there were probably several opportunities for support of self-management that we did not observe. Another limitation is the lack of observations and interviews with other healthcare professionals, such as physiotherapists and nutritionists, who might have been involved in patient discussions on lifestyle advice. Third, as no follow-up interviews were conducted with patients after arriving home, this study cannot draw any conclusions on the actual level of understanding or execution of self-management activities post-discharge. Lastly, we did not specifically target next-of-kin in the interviews, which may have caused us to miss important information on the understanding of the discharge information.

## Conclusion

Much resources and efforts are put into preparing for hospital discharge. Coordinators, going-home teams and RNs facilitate social service communications and practical details, whereas physicians gather all pieces of information from healthcare professionals to write plain-language discharge letters. The giving of oral information at hospital discharge follows the written discharge letter, which gives thorough information, especially when accompanied by the professionals’ use of simple pedagogical initiatives. Despite this, the discharge encounter seems to be ill-suited to actually help patients manage their post-discharge needs at home. Patients are stressed and focused on going home, and the information given has a mainly retrospective focus. A focus seems to be needed on shared understanding or availability of either in-hospital or out-hospital teams to help address patient motivation and skills going forward.

## Reviewers

Anna Bråndal Reg. Physiotherapist, PhD Department of public health and clinical medicine at Umeå University, Umeå, Sweden.

Gavin Daker-White, Research Fellow, NIHR Greater Manchester Patient Safety Translational Research Centre (Greater Manchester PSTRC), Division of Population Health, Health Services Research & Primary Care, School of Health Sciences, Faculty of Biology, Medicine & Health, University of Manchester, Manchester, UK.

Dagrunn Nåden Dyrstad, Associate Professor, Faculty of Health Studies, University of Stavanger, Norway.

## References

[1] Davis K (2010). A New Era in American Health Care: Realizing the Potential of Reform. The Commonwealth Fund.

[2] Jencks SF, Williams MV, Coleman EA (2009). Rehospitalizations among patients in the Medicare fee-for-service program. The New England journal of medicine.

[3] Leppin AL, Gionfriddo MR, Kessler M, Brito JP, Mair FS, Gallacher K (2014). Preventing 30-Day Hospital Readmissions: A Systematic Review and Meta-analysis of Randomized Trials. JAMA internal medicine.

[4] Arora VM, Prochaska ML, Farnan JM, D’Arcy MJt, Schwanz KJ, Vinci LM (2010). Problems after discharge and understanding of communication with their primary care physicians among hospitalized seniors: a mixed methods study. Journal of hospital medicine: an official publication of the Society of Hospital Medicine.

[5] Forster AJ, Murff HJ, Peterson JF, Gandhi TK, Bates DW (2003). The incidence and severity of adverse events affecting patients after discharge from the hospital. Annals of Internal Medicine.

[6] Moore C, Wisnivesky J, Williams S, McGinn T (2003). Medical errors related to discontinuity of care from an inpatient to an outpatient setting. Journal of General Internal Medicine.

[7] Barlow J, Wright C, Sheasby J, Turner A, Hainsworth J (2002). Self-management approaches for people with chronic conditions: a review. Patient Educ Couns.

[8] Batalden M, Batalden P, Margolis P, Seid M, Armstrong G, Opipari-Arrigan L, Hartung H (2015). Coproduction of healthcare service. BMJ quality & safety.

[9] Begum N, Donald M, Ozolins IZ, Dower J (2011). Hospital admissions, emergency department utilisation and patient activation for self-management among people with diabetes. Diabetes Res Clin Pract.

[10] Mitchell SE, Gardiner PM, Sadikova E, Martin JM, Jack BW, Hibbard JH (2014). Patient activation and 30-day post-discharge hospital utilization. Journal of general internal medicine.

[11] Shively MJ, Gardetto NJ, Kodiath MF, Kelly A, Smith TL, Stepnowsky C (2013). Effect of patient activation on self-management in patients with heart failure. J Cardiovasc Nurs.

[12] Kommuri NV, Johnson ML, Koelling TM (2012). Relationship between improvements in heart failure patient disease specific knowledge and clinical events as part of a randomized controlled trial. Patient Educ Couns.

[13] Dharmarajan K, Hsieh AF, Kulkarni VT (2015). Trajectories of risk after hospitalization for heart failure, acute myocardial infarction, or pneumonia: retrospective cohort study. Bmj.

[14] Patientlag, Svensk författningssamling 2014: 821 (2015). [Patient Act, Swedish regulations no. 2014: 821]. http://www.riksdagen.se/sv/dokument-lagar/dokument/svensk-forfattningssamling/patientlag-2014821_sfs-2014-821.

[15] NHS England (2014). Five year forward view. https://www.england.nhs.uk/wp-content/uploads/2014/10/5yfv-web.pdf.

[16] Hansen LO, Young RS, Hinami K, Leung A, Williams MV (2011). Interventions to reduce 30-day rehospitalization: a systematic review. Annals of internal medicine.

[17] Gonçalves-Bradley DC, Lannin NA, Clemson LM, Cameron ID, Shepperd S (2016). Discharge planning from hospital. Cochrane Database Syst Rev.

[18] Daker-White G, Hays R, McSharry J, Giles S, Cheraghi-Sohi S, Rhodes P (2015). Blame the Patient, Blame the Doctor or Blame the System? A Meta-Synthesis of Qualitative Studies of Patient Safety in Primary Care. PLoS One.

[19] Hesselink G, Flink M, Olsson M, Barach P, Dudzik-Urbaniak E, Orrego C (2012). Are patients discharged with care? A qualitative study of perceptions and experiences of patients, family members and care providers. BMJ quality & safety.

[20] Horwitz LI, Moriarty JP, Chen C, Fogerty RL, Brewster UC, Kanade S (2013). Quality of discharge practices and patient understanding at an academic medical center. JAMA internal medicine.

[21] Makaryus AN, Friedman EA (2005). Patients’ understanding of their treatment plans and diagnosis at discharge. Mayo Clin Proc.

[22] Ziaeian B, Araujo KL, Van Ness PH, Horwitz LI (2012). Medication reconciliation accuracy and patient understanding of intended medication changes on hospital discharge. Journal of general internal medicine.

[23] National Board of Health and Welfare in Sweden (2005). Socialstyrelsens föreskrifter om samverkan vid in- och utskrivning av patienter i sluten vård. [Regulations on collaboration at admission and discharge for inpatient care]. Socialstyrelsen.

[24] Flink M, Ohlen G, Hansagi H, Barach P, Olsson M (2012). Beliefs and experiences can influence patient participation in handover between primary and secondary care—a qualitative study of patient perspectives. BMJ quality & safety.

[25] Howard-Anderson J, Busuttil A, Lonowski S, Vangala S, Afsar-Manesh N (2016). From discharge to readmission: Understanding the process from the patient perspective. Journal of hospital medicine : an official publication of the Society of Hospital Medicine.

[26] Malterud K, Siersma VD, Guassora AD (2015). Sample Size in Qualitative Interview Studies: Guided by Information Power. Qualitative health research.

[27] Graneheim UH, Lundman B (2004). Qualitative content analysis in nursing research: concepts, procedures and measures to achieve trustworthiness. Nurse education today.

[28] Choudhry AJ, Baghdadi YM, Wagie AE, Habermann EB, Heller SF, Jenkins DH (2016). Readability of discharge summaries: with what level of information are we dismissing our patients?. American journal of surgery.

[29] World Health Organization (2003). ADHERENCE TO LONG-TERM THERAPIES Evidence for action. http://apps.who.int/iris/bitstream/10665/42682/1/9241545992.pdf.

[30] Roter DL, Hall JA, Merisca R, Nordstrom B, Cretin D, Svarstad B (1998). Effectiveness of interventions to improve patient compliance: a meta-analysis. Med Care.

[31] Fisher WA, Fisher JD, Harman J, Suls J, Wallston KA (2003). The Information–Motivation– Behavioral Skills Model: A General Social Psychological Approach to Understanding and Promoting Health Behavior. Social Psychological Foundations of Health and Illness: Blackwell Publishing Ltd.

[32] Greysen SR, Harrison JD, Kripalani S, Vasilevskis E, Robinson E, Metlay J (2016). Understanding patient-centred readmission factors: a multi-site, mixed-methods study. BMJ quality & safety.

[33] Ekstedt M, Aase K, Schibevaag L (2016). Cooping with complexity – Sensemaking in spezialised home care. Researching Patient Safety and Quality in Healthcare: A Nordic Perspective.

[34] Flemming D, Hubner U (2013). How to improve change of shift handovers and collaborative grounding and what role does the electronic patient record system play? Results of a systematic literature review. Int J Med Inform.

[35] White M, Garbez R, Carroll M, Brinker E, Howie-Esquivel J (2013). Is “teach-back” associated with knowledge retention and hospital readmission in hospitalized heart failure patients?. J Cardiovasc Nurs.

[36] Black P, Harrison C, Lee C, Marshall B, Wiliam D (2007). Assessment for learning – putting it into practice.

[37] Lin R, Gallagher R, Spinaze M, Najoumian H, Dennis C, Clifton-Bligh R (2014). Effect of a patient-directed discharge letter on patient understanding of their hospitalisation. Internal medicine journal.

[38] Crocker JB, Crocker JT, Greenwald JL (2012). Telephone follow-up as a primary care intervention for postdischarge outcomes improvement: a systematic review. The American journal of medicine.

[39] Mistiaen P, Poot E (2006). Telephone follow-up, initiated by a hospital-based health professional, for postdischarge problems in patients discharged from hospital to home. Cochrane Database Syst Rev.

[40] Braun E, Baidusi A, Alroy G, Azzam ZS (2009). Telephone follow-up improves patients satisfaction following hospital discharge. European journal of internal medicine.

